# High level of polarized engraftment of porcine intrahepatic cholangiocyte organoids in decellularized liver scaffolds

**DOI:** 10.1111/jcmm.17510

**Published:** 2022-08-26

**Authors:** Melanie Krüger, Roos‐Anne Samsom, Loes A. Oosterhoff, Monique E. van Wolferen, Hans S. Kooistra, Niels Geijsen, Louis C. Penning, Linda M. Kock, Pilar Sainz‐Arnal, Pedro M. Baptista, Bart Spee

**Affiliations:** ^1^ Department of Clinical Sciences, Faculty of Veterinary Medicine Utrecht University Utrecht The Netherlands; ^2^ Department of Anatomy and Embryology Leiden University Medical Center Leiden The Netherlands; ^3^ LifeTec Group BV Eindhoven The Netherlands; ^4^ Department of Biomedical Engineering Eindhoven University of Technology Eindhoven The Netherlands; ^5^ Laboratory of Organ Bioengineering and Regenerative Medicine Health Research Institute of Aragon (IIS Aragon) Zaragoza Spain

**Keywords:** bioengineering, cell polarity, livers, organoids, swine, tissue scaffolding

## Abstract

In Europe alone, each year 5500 people require a life‐saving liver transplantation, but 18% die before receiving one due to the shortage of donor organs. Whole organ engineering, utilizing decellularized liver scaffolds repopulated with autologous cells, is an attractive alternative to increase the pool of available organs for transplantation. The development of this technology is hampered by a lack of a suitable large‐animal model representative of the human physiology and a reliable and continuous cell source. We have generated porcine intrahepatic cholangiocyte organoids from adult stem cells and demonstrate that these cultures remained stable over multiple passages whilst retaining the ability to differentiate into hepatocyte‐ and cholangiocyte‐like cells. Recellularization onto porcine scaffolds was efficient and the organoids homogeneously differentiated, even showing polarization. Our porcine intrahepatic cholangiocyte system, combined with porcine liver scaffold paves the way for developing whole liver engineering in a relevant large‐animal model.

## 1 INTRODUCTION

Liver diseases impose a great burden on our society, in terms of suffering and death as well as economically, and in most countries, it is predicted to increase even further.^1^, ^2^, ^3^ These diseases such as drug induced liver injury,^4^ alcoholic liver disease,^5^ liver cancer,^6^ non‐alcoholic fatty liver disease^7^ and others together account for 2 million deaths per year worldwide. In Europe alone, more than 5500 liver transplants are performed each year, which is the only available treatment for end stage liver disease at this moment.^8^, ^9^ However, due to insufficient amounts of donor organs, 18% of people listed for a liver transplantation in Europe died before receiving a transplant.^10^ Therefore, an alternative to liver transplantation is urgently needed.

One such alternative is to decellularize livers that are discarded for transplantation, but provide a biological optimal scaffold for transplanted cells, such as intrahepatic cholangiocyte (ICOs).^11^, ^12^, ^13^, ^14^, ^15^, ^16^, ^17^ This native extracellular matrix (ECM) promotes cell differentiation, proliferation, attachment and cell migration.^15^, ^18^, ^19^ Moreover, liver‐ECM maintains hepatocyte function, possibly due to the intact three‐dimensional structure.^13^, ^20^ Thus, far recellularization of decellularized liver ECM with reimplantation has been performed in rodents,^21^, ^22^ providing proof‐of‐principle, but a larger animal model is needed in preclinical‐studies.^23^, ^24^, ^25^, ^26^

Pigs present a very suitable model in this respect due to a physiology that is very close to human,^27^ and decellularized porcine scaffolds can be repopulated at a low degree with rat/human liver organoids.^15^, ^28^, ^29^ This low engraftment was possibly caused by the species differences between donor cells in the decellularized ECM‐scaffold, as ideally autologous cells should be used.^22^ Even more importantly, porcine progenitor cells can help advance this preclinical research towards human patients as they represent a more human‐relevant animal model. The lack of established porcine ICOs impeded this research so far.

Therefore, the aim of this study was to establish ICO culture from porcine adult stem cells and repopulate porcine decellularized liver scaffolds with these organoids. This would pave the way for whole organ bioengineering that can be used as an alternative for organ donation in the future.

## 2 MATERIALS AND METHODS

### 2.1 Isolation porcine liver cells and culture hepatic organoids

Livers from four individual pigs were derived from fresh slaughterhouse material, placed in cooled DMEM/F12 media (Gibco™, Thermo Fisher Scientific, Waltham, MA, USA) and transported to the laboratory on ice. Porcine liver tissue was cut down into small tissue fragments of 2–3 mm^3^ using sterile blades and petri dishes and frozen down in dimethyl sulphoxide (DMSO)‐based freezing medium (Thermo Fisher Scientific, Waltham, MA, USA) until further processing. Before cell isolation, the tissue was thawed rapidly to 37°C in a water bath. After further mechanical dissection into fragments of 1–2 mm^3^, liver tissue was enzymatically digested in DMEM (Life Technologies, Carlsbad, CA, USA) containing 0.3 mg/ml type II collagenase (Life Technologies) and 0.3 mg/ml dispase (Life Technologies) at 37°C until biliary duct fragments appeared in the suspension observed through the microscope (2–4 h). The isolated cells and biliary duct fragments were mixed with Matrigel™ (Corning, Glendale, AZ, USA) and plated in a 24‐wells plate in drops of 50 μl. Porcine ICOs were grown in expansion medium (EM) based on advanced DMEM/F12 (Life Technologies) containing 1% (v/v) Glutamax (Life Technologies), 1% (v/v) Penicillin–Streptomycin (Life Technologies), 1% (v/v) HEPES (Life Technologies), 30% (v/v) Wnt3a‐conditioned medium (prepared as in^30^), 1% (v/v) N2 (Invitrogen, Waltham, MA, USA), 10 mM nicotinamide (Sigma‐Aldrich, Saint Louis, MO, USA), 2% (v/v) B27 without vitamin A (Invitrogen), 10% (v/v) R‐spondin conditioned medium (Rspo1‐Fc expressing cell line kindly gifted by Calvin J. Kuo, Stanford University, CA, USA), 1.25 mM N‐acetylcysteine (Sigma‐Aldrich), 10 μM Y‐27632 (Selleckchem, Munich, Germany), 5 μM A83‐01 (Tocris Bioscience, Bristol, UK), 50 ng/ml human epidermal growth factor (EGF, Invitrogen), 0.1 μg/ml human noggin (Peprotech, London, UK), 0.1 μg/ml fibroblast growth factor 10 (FGF10, Peprotech), 10 nM gastrin (Sigma‐Aldrich), 25 ng/ml hepatocyte growth factor (HGF, Peprotech), 10 μM forskolin (Sigma‐Aldrich), and 1% (v/v) primocin (Invitrogen). For an overview of the medium composition please see Table S2.

### 2.2 Porcine intrahepatic cholangiocyte organoid differentiation

Prior to differentiation, porcine ICOs from 4 donors (*n* = 3 per donor) in the same passage number (p4) were cultured in EM with 25 ng/ml BMP7 (Peprotech) for 5 days. Differentiation medium (DM) was based on advanced DMEM/F12 (Life Technologies) containing 1% (v/v) Glutamax (Life Technologies), 1% (v/v) Penicillin–Streptomycin (Life Technologies), 1% (v/v) HEPES (Life Technologies), 1% (v/v) N2 (Invitrogen), 2% (v/v) B27 without vitamin A (Invitrogen), 1.25 mM N‐acetylcysteine (Sigma‐Aldrich), 5 μM A83‐01 (Tocris Bioscience), 50 ng/mL human epidermal growth factor (EGF, Invitrogen), 0.1 μg/ml human noggin (Peprotech), 0.1 μg/ml FGF19 (R&D Systems, Minneapolis, MN, USA), 10 nM gastrin (Sigma‐Aldrich), 25 ng/ml hepatocyte growth factor (HGF, Peprotech), 30 μM dexamethasone (Sigma‐Aldrich), 10 μM DAPT (y‐secretase inhibitor, Selleckchem), and 25 ng/ml BMP7 (Peprotech), and 1% (v/v) primocin (Gentaur, Kampenhout, Belgium). Differentiation medium was refreshed every 3 days until the end of differentiation culture (Day 5 and 9).

### 2.3 De‐ and recellularization

The process of porcine liver decelluarization and recellularization is schematically depicted in Figure 3A.

#### 2.3.1 Decellularization of porcine livers

Decellularization of porcine livers was performed according to the previously published protocol by Pla‐Palacin et al.^31^ In short, whole livers are dissected from pig cadavers and portal vein and hepatic artery are cannulated and subsequently perfused with alternating charges of distilled water and decellularization solution containing Triton X‐100. The decellularized livers were then cut into circular discs of 5 mm diameter and 200 μm height.

#### 2.3.2 Recellularization of liver scaffolds

Before reseeding, the scaffolds were incubated for 1 h at 37°C with 200 μl EM with 25 ng/ml BMP7 in a humidified incubator at 37°C, 5% CO_2_ and 21% oxygen. After the incubation the medium was removed, and the scaffolds were placed in an incubator at 37°C for 1 h to dry. Each scaffold was seeded with 200 000 cells as organoid fragments in 10 μl EM with the addition of 25 ng/ml BMP7 in a 96‐well plate. After 1 h incubation in an incubator at 37°C, 250 μl EM with 25 ng/ml BMP7 was added to each well. The EM was refreshed twice a day. After 2 days, the scaffolds were transferred to a 24 well plate and the medium was changed to DM. The DM was refreshed every 2–3 days until the end of differentiation at Day 5, which has proven to be the best timepoint based on preliminary experiments (data not shown).

### 2.4 Functional analyses

#### 2.4.1 Metabolic activity

To test the metabolic activity of the organoids grown in expansion medium, Alamar Blue (Invitrogen) was diluted in DMEM/F12 without phenol red (1:20, Life Technologies) and incubated with the samples (*n* = 3 per donor) for 2 h at 37°C. The fluorescence was measured with a spectrophotometer (Fluoroskan Ascent FL, Thermo Fisher Scientific, Waltham, MA, USA) at 544/570 nm. The Alamar Blue assay was performed on all four donors in passage (p2) and the intensity was measured at Days 0, 2, 6 and 8 after plating.

#### 2.4.2 Gene expression

To get an overview of the gene expression profile of porcine ICOs in EM (in Matrigel™) and DM conditions (in Matrigel™ and discs), RNA was isolated from organoids (*n* = 3 per donor, condition and time point) using the RNAeasy Micro/Mini Kit (Qiagen, Hilden, Germany) following manufacturer's instructions. Subsequently, quality and quantity were defined with Nanodrop equipment (DS‐11 Spectrophotometer, DeNovix). cDNA was prepared using the iScript cDNA Synthesis Kit (Bio‐Rad, Hercules, CA). Quantitative PCR was performed on Bio‐Rad MyiQ Cycler using SYBRgreen 10 supermix (Bio‐Rad). Quantification of target gene expression was normalized to the geometric mean of the reference genes glyceraldehyde‐3‐phosphate dehydrogenase (GAPDH), ribosomal protein S19 (RPS19), hydroxymethylbilane synthase (HMBS) and tyrosine‐3‐monooxygenase/tryptophan 5‐monooxygenase activation protein zeta (YWHAZ), as required under MIQE‐precise.^32^ The delta‐Cq method was used for analysis. Selected target genes were HNF1B (Hepatocyte nuclear factor 1 homeobox B), G‐protein coupled receptor 5 (LGR5), albumin (ALB), cytochrome P450, family 3, subfamily alpha, polypeptide 22 (CYP3A22), hepatocyte nuclear factor 4 alpha (HNF4A), transferrin (TF), fumarylacetoacetate hydrolase (FAH) and transthyretin (TTR). The primer details are shown in Table 1.

**TABLE 1:** Primer information for porcine specific qRT‐PCR

Gene	Forward Primer 5′ ➔ 3′	Reverse Primer 5′ ➔ 3′	NCBI reference sequence
GAPDH	CTGCCCAGAACATCATCCC	CAGTGAGCTTCCCGTTGAG	NM_001206359.1
RPS19	AAAGAAACGGTGTCATGCCC	AGGCCTTTCCCATCTTGGT	XM_005655906.3
HMBS	GATGGGCAACTCTACCTGAC	CAAGCTGTGGGTCATCCTC	NM_001097412.1
YWHAZ	CAAAGACAGCATTTGATGAAGCC	ATCTCCTTGGGTATCCGATGTC	NM_001315726.1
HNF1B	CCACCAACAAGAAGATGCGT	CAAACACTCTGCTCTGTTGC	NM_213956.1
LGR5	CAACTTGCAGAAGATTTACCCAG	GCTAAATTCAGAGATCGGAGG	NM_001315762.1
ALB	CGCTCATAGTTCGTTACACC	CTTACAACACCTAGAGCCCA	NM_001005208.1
CYP3A22	CATCAACACGAAAGAAATCTTTGGG	GTCTCGTGGGTTGTTGAGG	NM_001195509.1
HNF4A	CTTCTTTGACCCAGATGCC	GTCGTTAGATGTAATCCTCCAG	NM_001044571.1
TF	GCCATCAGGGATAAAGAAGCA	GCCCATAGAACTCTGCCAC	NM_001244653.1
FAH	CCAAGATGTCTTTGATCAGCCA	CCGAAGTTCTGTGTCATCTCTG	XM_003356648.4
TTR	AATATGCAGAGGTTGTGTTCACAG	CTGTGGTGGAGTAAGAGTAGGG	NM_214212.1

Abbreviations: ALB, Albumin; CYP3A22, Cytochrome P450 family 3, subfamily alpha, polypeptide 22; FAH, Fumarylacetoacetate hydrolase; GAPDH, Glyceraldehyde‐3‐phosphate dehydrogenase; HMBS, Hydroxymethylbilane synthase; HNF4A, Hepatocyte nuclear factor 4 alpha; LGR5, G‐protein coupled receptor 5; RPS 19, Ribosomal protein S19; TF, Transferrin; TTR, Transthyretin; YWHAZ, Tyrosine‐3‐monooxygenase/tryptophan 5‐monooxygenase activation protein zeta.

#### 2.4.3 Immunofluorescence

Liver tissue biopsies and organoids harvested from Matrigel™ (*n* = 3 per donor and condition) on Day 5 were fixed in 4% (w/v) phosphate buffered formaldehyde (PFA) with 0.1% (w/v) eosin for 1 h and embedded in 2.5% (w/v) agar (BD). The embedded organoids were dehydrated with gradient ethanol and xylene (Klinipath, Duiven, The Netherlands), embedded in paraffin (Leica, Wetzlar, Germany) and sectioned into 4 μm thin sections. First, the slides were incubated at 60°C for 10 min. Sections were stained for keratin 19 (K19), albumin (ALB), multidrug resistance protein 1 (MDR1) and proliferation marker Ki‐67 for all conditions (EM, DM, discs); for tight junction protein 1 (ZO1) in DM and discs; and hepatocyte nuclear factor 4 alpha (HNF4A), keratin 18 (K18). For each antibody, the antigen retrieval method, antibody dilution and incubation times are summarized in Table 2. Slides were analysed using a Leica SPEII fluorescent microscope.

**TABLE 2:** Antibody information for immunofluorescent staining

Antibody	Dilution	Incubation time	Antigen retrieval	Washing buffer	Secondary antibody	Supplier
K19	1:50	Overnight	Citrate 98°C for 30 min	PBS/Tween 0.1%	AF488 goat‐anti‐rabbit 1:200	Abcam (ab76539)
ALB	1:1500	Overnight	Citrate 98°C for 30 min	PBS/Tween 0.1%	AF488 goat‐anti‐mouse 1:200	Sigma (A6684)
HNF4A	1:500	Overnight	Tris/EDTA 98°C for 30 min	TBS/Triton 0.2%	AF488 goat‐anti‐rabbit 1:200	Santa Cruz (sc‐8987)
Ki‐67	1:50	Overnight	Citrate	PBS/Tween 0.1%	AF488 goat‐anti‐rabbit 1:200	Dako (M7240)
ZO‐1	1:250	Overnight	Pepsin 0.4% in 0.2 N HCl 20 min at 37°C	PBS/Tween 0.1%	AF488 goat‐anti‐rabbit 1:200	Invitrogen (40–2300)
K18	1:100	Overnight	Citrate 98°C 3 for 0 min	PBS/Tween 0.1%	AF488 goat‐anti‐mouse 1:200	Santa Cruz (sc‐51,582)
MDR1	1:200	Overnight	Tris/EDTA 98°C for 30 min	PBS/Tween 0.1%	AF488 goat‐anti‐rabbit 1:200	Novus bio (NBP1‐90291)

Abbreviations: ALB, Albumin; HNF4A, Hepatocyte nuclear factor 4 alpha; K18, Keratin 18; K19, Keratin 19; Ki 67, Proliferation marker; MDR1, Multidrug resistance protein 1.; ZO1, Tight junction protein 1.

#### 2.4.4 Clinical chemistry

For measurement of the expression of liver transaminases and albumin production in differentiated organoids, cells were lysed in MilliQ (MilliPore, Burlington, MA, USA) at Day 5 and stored at −20°C (*n* = 3 for each donor and timepoint). Albumin (ALB), Aspartate Transaminase (ASAT), Lactate dehydrogenase (LDH) and Glutamate Dehydrogenase (GLDH) were measured using the DxC‐600 Beckman (Backman Coulter, Brea, CA, USA) standard protocols, and the values were corrected for the total protein counts.

#### 2.4.5 Engraftment efficiency

Engraftment percentage was determined by analysing representative 4× images of decellularized discs for each donor that were stained with Haematoxylin and Eosin with ImageJ software. The total scaffold area was determined after which the areas without cells were subtracted.

#### 2.4.6 Statistics

All statistics have been performed with GraphPad Prism 8.0 and all data is normally distributed. Data are shown as mean ± SD. Viability data was analysed using a mixed‐model with Tukey's multiple comparison test, with examination of residual plot for fit and Q‐Q plot for normality. Enzyme data was analysed using 2‐way repeated measures anova with Sidak's multiple comparisons test. A *p*‐value of *p* < 0.05 was considered statically significant.

## 3 RESULTS

### 3.1 Organoid expansion

**FIGURE 1:**
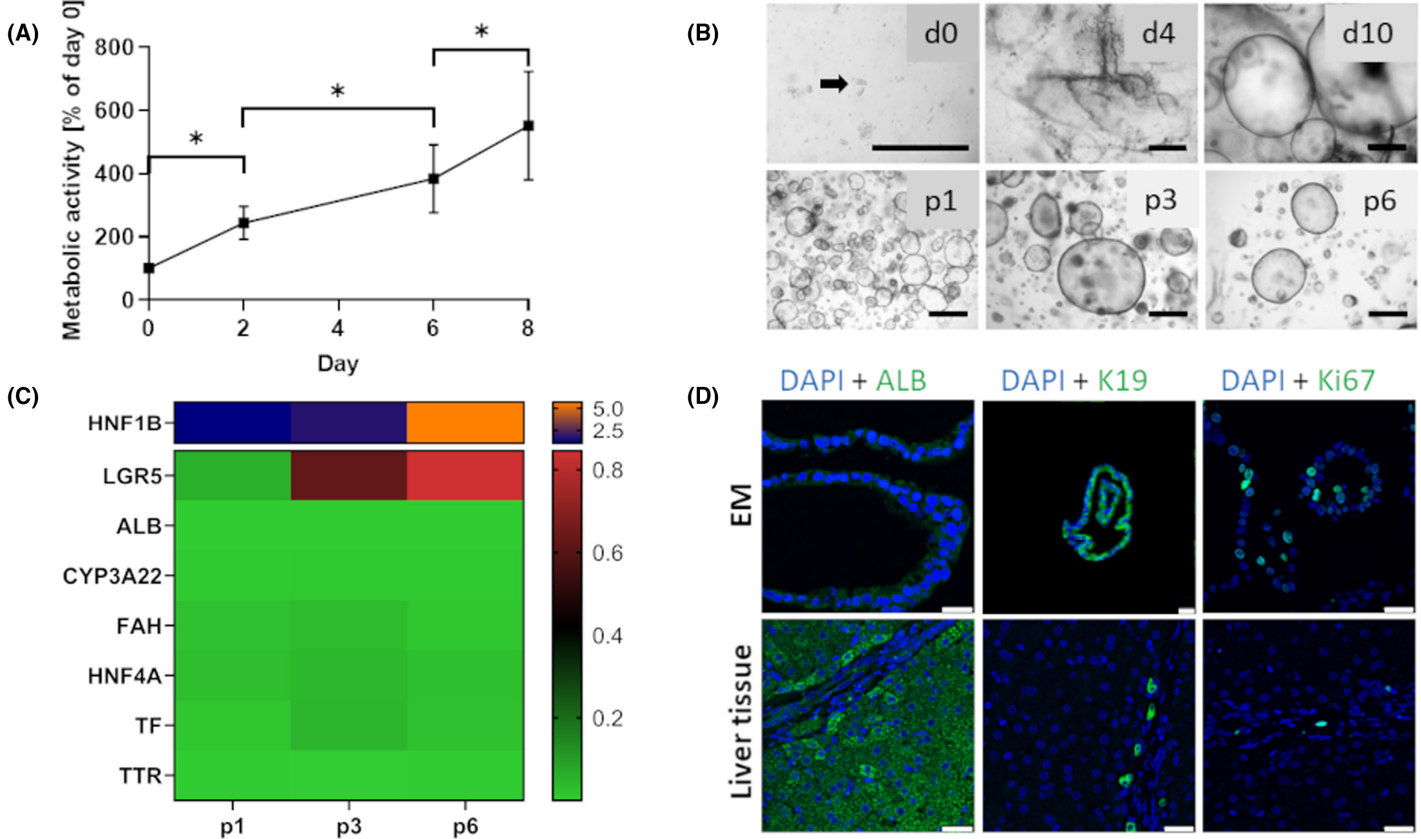
(A) Metabolic activity according to Alamar Blue assay of porcine ICOs over 8 days of culture in EM media as percentage of Day 0. Data depicted as mean ± SD (*n* = 4), *p < 0.0001. (B) Establishment of organoid culture from porcine liver tissue samples in representative phase‐contrast images of duct isolation and organoid culture. After digestion, biliary ducts fragments were observed in culture (d0, arrow). Ducts were cultured in Matrigel™ and defined medium. After 4 days in culture (d4), spherical structures appeared that grew out to organoids within 10 days (d10). Representative phase‐contrast images of porcine ICOs in different passages (p1, p3 and p6). Scale bars represent 500 μm. (C) Heatmap showing gene expression relative to liver over 6 passages of organoid culture (median of donors, *n* = 3 per donor) with increased HNF1B (Hepatocyte nuclear factor 1 homeobox B) and LGR5 (G‐protein coupled receptor 5) expression and stable ALB (Albumin), CYP3A22 (Cytochrome P450, family 3, subfamily alpha, polypeptide 22), FAH (Fumarylacetoacetate hydrolase), HNF4A (Hepatocyte nuclear factor 4 alpha), TF (Transferrin) and TTR (Transthyretin) expression. (D) Representative immunofluorescent staining for ALB, K19 (Keratin 19), Ki67 (Proliferation marker) expression (in green) and nuclei (DAPI in blue) for organoids in EM (Expansion medium) conditions after day 5–7 and native liver tissue. Scale bars represent 25 μm

The cells derived from porcine liver tissue samples were cultured in Matrigel™ and biliary duct fragments could be observed in the hydrogel scaffold (see Figure 1B). Over the next days of culture in EM media the formation of cyst‐like spherical structures and their growth could be observed and their morphology stayed stable until at least passage 6 (see Figure 1B). Organoid growth was also shown by metabolic activity measurement (see Figure 1A), where activity significantly increased from day to day up to 451% compared with the start of culture (*p* < 0.0001). In Figure 1C, gene expression (values in Table S1) shows that with increasing passage the expression level of stem cell marker Leucine Rich Repeat Containing G‐Protein Coupled Receptor 5 (LGR5) and Hepatocyte nuclear factor 1 homeobox B (HNF1B) slightly increased, whereas expression of all other hepatocyte specific markers such as Hepatocyte nuclear factor 4 alpha (HNF4A), Albumin (ALB), Transferrin (TF), Transthyretin (TTR), Fumarylacetoacetate hydrolase (FAH), Cytochrome P450 3A22 (CYP3A22, porcine analogue to human CYP3A4) remained stable over time.

Staining for hepatocyte marker albumin as well as polarization marker Multidrug resistance 1 (MDR1) shows that there was already slight presence of the protein in the expanding organoids, although less compared with native liver tissue (Figures 1D and S1). In contrast, the organoids stained strongly for the ductular marker K19 and the proliferation marker Ki67 stained roughly 30 percent of the cells within the organoid showing continued expansion (Figure 1D).

### 3.2 Organoid differentiation

**FIGURE 2:**
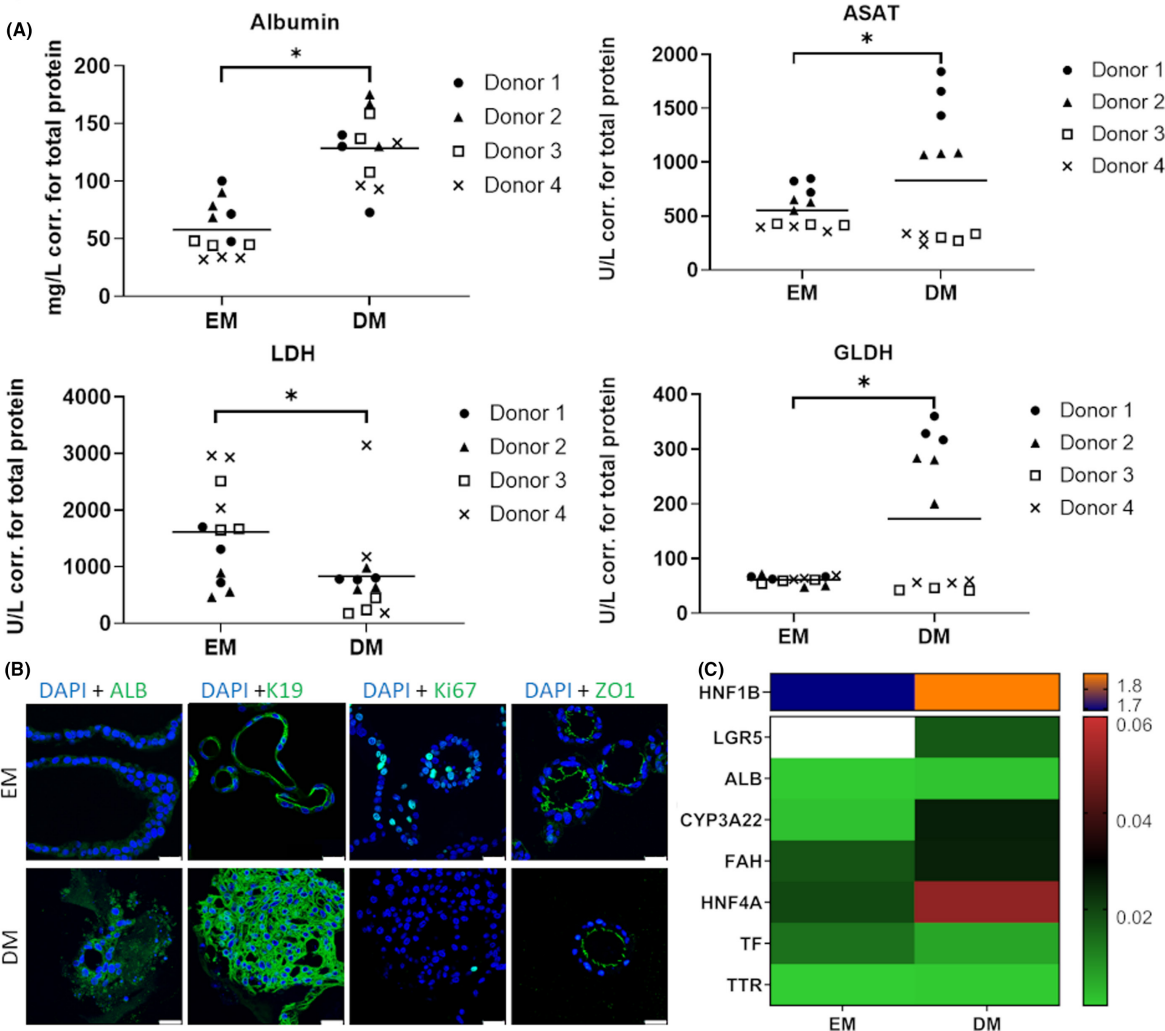
(A) Functional read‐out in albumin secretion and enzyme activity (ASAT (Aspartate transaminase), LDH (Lactate dehydrogenase) and GLDH (Glutamate dehydrogenase)) corrected for total protein after 5 days in DM (Differentiation medium) compared with EM (Expansion medium). Data depicted as grand mean (*n* = 4), *p < 0.05, (B) Representative immunofluorescent staining for hepatocyte marker ALB (Albumin), ductular marker K19 (Keratin 19), proliferation marker (Ki67), polarization marker ZO1 (Tight junction protein 1) expression (all in green) and nuclei (DAPI in blue) for organoids in DM conditions after Day 5 compared with EM conditions. Albumin and K19 are increased in DM, whereas Ki67 is decreased and ZO1 stable. Scale bars represent 25 μm. (C) Heatmap showing gene expression relative to liver after 5 days of culture for DM conditions compared with EM conditions (median of donors, *n* = 3 per donor) with decreased expression of LGR5 (G‐protein coupled receptor 5) (EM value above the top end of the scale) and TF (Transferrin), stable ALB and TTR (Transthyretin) expression, and increased expression of HNF1B (Hepatocyte nuclear factor 1 homeobox B), CYP3A22 (Cytochrome P450, family 3, subfamily alpha, polypeptide 22), FAH (Fumarylacetoacetate hydrolase) and HNF4A (Hepatocyte nuclear factor 4 alpha)

After differentiation of the porcine ICOs for 5 days, the grand mean of the albumin level had significantly increased by 71 mg/L (*p* < 0.0001) in DM compared with EM (see Figure 2A). Multiple comparisons show that for donor 3 the albumin level has even increased by 89 mg/L (*p* = 0.0002) and by 78 mg/L (*p* = 0.0005) for donor 2. Equally, mean ASAT activity was significantly higher (278 U/L; *p* < 0.0001) in DM compared with EM despite some variation between donors that ranged from no significant changes amongst the medium conditions for donors 3 and 4 to an increase of 847 U/L (*p* < 0.0001) for donor 1. LDH activity levels were significantly lower after 5 days in differentiation medium (788 U/L; *p* = 0.013), which was mainly due to the large decrease of activity of donor 3 (1654 U/L; *p* = 0.041), as all other donors showed no significant difference between EM and DM levels. Mean GLDH activity after 5 days of culture in DM conditions compared with EM increased by 111 U/L (*p* < 0.0001) with as much as 270 U/L (*p* < 0.0001) and 198 U/L (*p* < 0.0001) for donors 1 and 2, respectively.

As opposed to EM conditions, albumin was much more strongly expressed after differentiation of the organoids as demonstrated by the immunofluorescent staining in Figure 2B. Similarly, keratin 19 was more widespread around the nuclei compared with organoids under expansion conditions. The proliferation marker Ki67 is visible in fewer cells of the differentiated organoids than in expanding specimen. Polarization marker MDR1 accumulated on the liminal side of the ICOs compared with EM conditions, where it was not present at all (Figure S1). The tight junction protein ZO1 accumulated similarly on the luminal side of the organoids in both EM and DM conditions.

In Figure 2C, the heatmap clearly illustrates differentiation by 46.4‐fold reduction of LGR5 expression in the differentiated organoids, as well as an increase in HNF1B (1.1‐fold), CYP3A22 (15.5‐fold), FAH (1.4‐fold) and HNF4A (2.6‐fold) expression. Both ALB and TTR expression showed no difference between these two conditions and TF was expressed slightly less in DM compared to EM.

### 3.3 Decellularized discs

**FIGURE 3:**
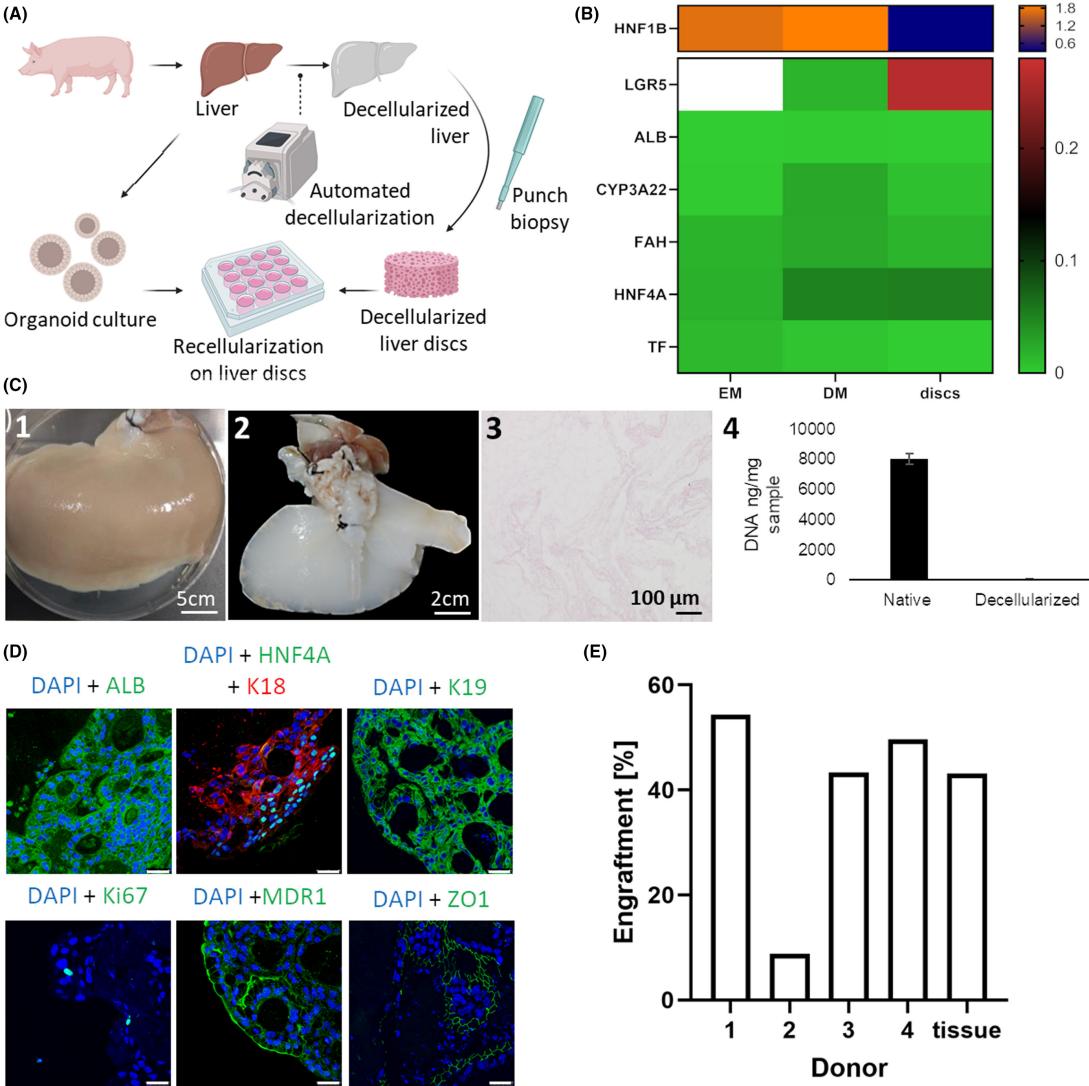
(A) Scheme demonstrating how porcine materials are used to tissue engineer livers from decellularized tissue and hepatic organoids. (B) Heatmap showing gene expression relative to liver after 5 days of culture for recellularized discs compared with EM (Expansion medium) and DM (differentiation medium) conditions (median of donors, *n* = 3 per donor) with increased expression of LGR5 (G‐protein coupled receptor 5) and HNF4A (Hepatocyte nuclear factor 4 alpha) in discs (EM value LGR5 off the top end of the scale) and decreased HNF1B (Hepatocyte nuclear factor 1 homeobox B), TF (Transferrin), stable ALB (Albumin) expression, and less CYP3A22 (Cytochrome P450, family 3, subfamily alpha, polypeptide 22), FAH (Fumarylacetoacetate hydrolase) expression compared with DM. (C) Porcine liver decellularization and characterization—(1) Native porcine liver (2) Decellularized porcine right liver lobe (3) Haematoxylin & Eosin staining showing full cellular ablation (4) DNA extraction from the decellularized liver tissue shows a clear reduction of the amount of DNA present when compared with native hepatic tissue. (D) Representative immunofluorescent staining for nuclei (DAPI in blue) and hepatocyte marker ALB, ductular marker K19 (Keratin 19), proliferation marker (Ki67), polarization markers MDR1 (Multidrug resistance protein 1), ZO1 (Tight junction protein 1) (all in green) and hepatocyte marker K18 (Keratin 18) (in red) expression for organoids in recellularized discs after Day 5. ALB, MDR1 and Ki67 show positive staining, K18 and K19 are highly expressed, and HNF4A only evident in part of the discs. Scale bars represent 25 μm

Successful decellularization of livers was confirmed by the lack of nuclei and DNA present (see Figure 3C), and exemplary images of native versus decellularized liver scaffolds can be seen in Figure 3C as well. The organoids seeded onto these scaffolds showed lower expression of stemness markers LGR5 compared with EM control as well as lower expression of HNF1B (see Figure 3B). All hepatic markers were expressed by organoids seeded onto scaffolds. As seen by similar expression levels of HNF4A, ALB, TF, TTR, FAH and CYP3A22 between ICO's on scaffolds and in DM, maturation of the hepatocyte‐like cells was at a comparable level.

The immunofluorescent stainings showed that the organoids infiltrated the scaffold with a high efficiency and proceeded to express several different markers (Figure 3D). Albumin showed a positive staining in the vast majority of the cells seeded onto scaffolds. The hepatic markers HNF4A and K18 stained positive in several areas of the scaffolds indicating a hepatic differentiation in specific zones within the scaffold. The location of MDR1 also showed that the cells polarized on the scaffolds and formed canaliculi between cells. Some proliferative cells were observed in the scaffold as determined by a positive nuclear staining for Ki67. Notably, organoids remained positive for ductular marker K19 on the scaffolds. Tight junction protein ZO1 was also positive throughout the scaffold indicating the creation of tight junctions between cells. The high engraftment efficiency, very similar to native tissue for most donors, can be seen in Figure 3E.

## 4 DISCUSSION

In this study, we show that ICOs can be successfully grown and differentiated from porcine liver tissue. Both the morphology as well as gene expression are stable over time and are both comparable to human ICOs grown in Matrigel™^33^, ^34^ and to the limited data on porcine ICOs.^27^ The ability of the porcine ICOs to differentiate was proven by the loss of stem cell and progenitor cell characteristics, increase in hepatocyte and cholangiocyte markers and functional assays that demonstrated increased liver enzyme activity, and epithelial polarity of the cells as shown by ZO1 localization.^35^ Notably, expression of HNF1B was very variable between donors, which might be due to different extraction locations of liver tissue and should therefore be taken into consideration in future experiments.^36^ Although most donors behaved similarly in the clinical chemistry assays performed, two donors showed less hepatic differentiation for ASAT and GLDH. At this stage it remains unclear what the discrepancy for these two measurements is, although one indication is given by the low engraftment efficiency for donor 2. The positive staining of K19 validated the presence of ductal cells,^37^ as did the increase of the transcription factor HNF1B only expressed by biliary epithelial cells.^38^ The discrepancy between the increased albumin protein levels and the constant albumin gene expression levels in differentiated compared with expanding cells could potentially be explained by the fact that albumin gene expression is known to be feedback regulated by the presence of albumin in serum.^39^

Notably, immunofluorescent staining for K18 was very high and was observed in proximity of HNF4A positive cells, which further confirms the transformation towards hepatocyte‐like cells.^40^ The high expression of K18 could be explained by the hypothesis that as these keratins protect hepatocytes against apoptosis, the cells on the discs are experiencing stress but are functional enough to activate counteractions and differentiate towards hepatocyte cell fate.^37^ The organoids had been fragmented before seeding onto the scaffold, but nevertheless proceeded to self‐organize as is evident by the polarization seen in MDR1 and ZO1 stainings.^35^ The formation of canaliculi as shown here by K19 and MDR1 positive staining has previously been unsuccessful when human foetal hepatocytes have been used to repopulate decellularized porcine scaffolds even after 13 days of perfusion time.^15^ It is possible that this is caused by cross‐species differences between scaffold and cell source.

Although differentiation was largely successful, the resulting cells do not exhibit the same characteristics as native hepatocytes or cholangiocytes as seen for example in the localized expression of HNF4A or the lack of liver transaminase production of some donors and further optimization/maturation is necessary. Additionally, animal derived components such as Matrigel™ and Wnt cannot be used in a clinical setting and must be replaced by alternatives such as other ECM‐mimicking hydrogels (cellulose nanofibril,^34^ gelPEG,^41^ Gelatin‐methacrylol or other hydrogels^42^) and Wnt‐surrogates.^43^

Additionally, medium compositions could be further optimized in future studies. In the present study, proliferation of organoids in expansion medium is mediated by R‐spondin that activates Wnt signalling and is therefore present in EM medium but absent in DM medium.^44^ Here, the combination of hepatocyte growth factor and dexamethasone drives maturation towards hepatocyte‐like cells,^45^ as do FGF19 and BMP7.^46^ Maturation could be enhanced by adding other compounds or ECM components during the differentiation such as Oncostatin M or fibronectin.^47^, ^48^To increase both cholangiocyte and hepatocyte differentiation, addition of collagen type I to the culture system have been proven to be of advantage.^44^, ^48^ Maturation of bile duct structures is achieved in the current study by utilizing the Notch inhibitor DAPT in DM medium, but biliary differentiation could be increased by addition of laminin and collagen IV.^48^

In this study, only small scaffold pieces were utilized to allow the cells to repopulate the ECM by diffusion which was very successful as seen by the engraftment percentage. However, in order to achieve complete population, it would be beneficial to use perfused liver scaffolds that make use of the native vascular network in the decellularized organ.^18^, ^28^, ^31^ Thereby, it is of advantage to use fragmented organoids that are capable of self‐organization as the whole organoids would be too large to pass through the smaller microvasculature of the liver and would also fail to repopulate the scaffold. Organoids, in particular porcine ones, possess the added advantage of being a representative model for disease modelling and drug research.^27^ In addition to the administration of cells to the scaffold, co‐culture of several cell‐types is needed for differentiation,^49^ but also to gain full functionality of a native liver including bile secretion. As seen by the decrease of HNF1B expression, also differentiation into cholangiocytes, and therefore, bile duct formation is not at the level as seen in the DM Matrigel condition, whereas differentiation into hepatocyte‐like cells was preferred as seen by the increase of HNF4A. Other required co‐culture cells include parenchymal and non‐parenchymal cells^50^ as well as vascular endothelial cells.^51^ Recently, a novel method to scale up ICO production based on human stem cells was presented,^40^ which if applied successfully to the porcine model could lead to the cell amounts necessary to engineer whole livers. This method allows 40‐fold expansion of organoids in spinner flasks in only 2 weeks as opposed to the regular 6‐fold expansion in static cultures and further aids in differentiation towards hepatocytes. Therefore, further research will focus on co‐culturing of several cell‐types, which ideally are grown in animal‐free media at large scale to enable clinical application. Furthermore, engraftment efficiency in larger scaffolds could be achieved by perfusion of the scaffolds with cell‐containing medium and further characterization of the ICO's as well as the recellularized scaffolds will be performed to extent the knowledge on the cholangiocyte fate and stemness of the cells.

## 5 CONCLUSION

The aim of this work was to establish ICOs from porcine adult stem cells, which has been successful as they did not only show stable liver stem cell characteristics over multiple passages, but also differentiated into hepatocyte‐ and cholangiocytes‐like cells. This differentiation was also possible on decellularized porcine liver scaffolds and the cells repopulated the liver discs, which has been very effective, and polarization even showed formation of canaliculi. This is an important step towards bioengineering whole liver tissue in a relevant large‐animal model. With further improvements of organoids and repopulation of scaffolds with multiple cell types, the presented model can aid in understanding and treating liver diseases but also make whole organ engineering possible for transplantation and alleviate the organ donor shortage.

## AUTHOR CONTRIBUTIONS

**Melanie Krüger:** Conceptualization (lead); data curation (lead); formal analysis (lead); investigation (lead); methodology (lead); visualization (lead); writing – original draft (lead). **Roos‐Anne Samsom:** Data curation (equal); formal analysis (equal); investigation (equal); visualization (equal); writing – original draft (equal). **Loes A. Oosterhoff:** Investigation (supporting). **Monique E. van Wolferen:** Investigation (supporting). **Hans S. Kooistra:** Funding acquisition (supporting); project administration (supporting); writing – review and editing (supporting). **Niels Geijsen:** Project administration (supporting); writing – review and editing (supporting). **Louis C Penning:** Project administration (supporting); writing – original draft (equal); writing – review and editing (equal). **Linda M. Kock:** Funding acquisition (supporting); project administration (supporting); writing – review and editing (supporting). **Pilar Sainz‐Arnal:** Resources (supporting); visualization (supporting). **Pedro M. Baptista:** Conceptualization (equal); funding acquisition (equal); project administration (equal); resources (equal); writing – original draft (equal); writing – review and editing (equal). **Bart Spee:** Conceptualization (equal); funding acquisition (equal); project administration (equal); resources (equal); writing – original draft (equal); writing – review and editing (equal).

## FUNDING INFORMATION

This work has received funding from the European Union's Horizon 2020 research and innovation programme under the Marie Sklodowska‐Curie grant agreement No 64268.

## CONFLICT OF INTEREST

The author(s) declare no potential conflicts of interest with respect to the research, authorship and/or publication of this article.

## Supporting information


**Table S1** **–S2**
Figure S1

## Data Availability

Data relevant to the study is included in the article. Any additional or detailed data can be requested by directly emailing the corresponding author.
