# Supplementary material for: High level of polarized engraftment of porcine intrahepatic cholangiocyte organoids in decellularized liver scaffolds
Source: J Cell Mol Med. 2022 Aug 26;26(19):4949–58. doi: 10.1111/jcmm.17510 (PMC9549510; doi:10.1111/jcmm.17510)
Supplement: Supplementary file 1 — Table S1 –S2 Figure S1 [file JCMM-26-4949-s001.docx]

# Supplementary Material

Supplementary Table 1: Relative gene expression to liver per condition (EM (expansion medium), DM (differentiation medium), p1,3,6 (passage), discs (recellularized scaffolds). LGR5 (G-protein coupled receptor 5), HNF1B (hepatocyte nuclear factor 1 homeobox B), ALB (Albumin), CYP3A22 (Cytochrome P450, family 3, subfamily alpha, polypeptide 22), HNF4A (Hepatocyte nuclear factor 4 alpha), TF (Transferrin), FAH (Fumarylacetoacetate hydrolase), TTR (Transthyretin),

| Marker | Condition | Relative gene expression to liver  [Median (interquartile range)] |
| --- | --- | --- |
| LGR5 | EM, p1  EM, p3  EM, p6 | 5.84 x10^-02^ (1.87 x10^-01^, 1.75 x10^-02^)  6.24 x10^-01^ (6.97 x10^-01^, 3.56 x10^-01^)  8.45 x10^-01^ (1.87 x10^+00^, 7.64 x10^-01^) |
|  | EM | 7.90 x10^-01^ (1.47 x10^+00^, 3.76 x10^-01^) |
|  | DM | 1.70 x10^-02^ (5.01 x10^-02^, 3.40 x10^-03^) |
|  | Discs | 2.64 x10^-01^ (1.60 x10^+00^, 4.26 x10^-02^) |
| HNF1B | EM, p1  EM, p3  EM, p6 | 1.03 x10^+00^ (7.49 x10^+01^, 6.71 x10^-01^)  1.69 x10^+00^ (5.24 x10^+01^, 1.24 x10^+00^)  5.72 x10^+00^ (1.32 x10^+01^, 3.51 x10^+00^) |
|  | EM | 1.69 x10^+00^ (2.20 x10^+00^, 8.14 x10^-01^) |
|  | DM | 1.88 x10^+00^ (2.24 x10^+00^, 1.33 x10^+00^) |
|  | Discs | 3.25 x10^-01^ (3.38 x10^-01^, 2.83 x10^-01^) |
| ALB | EM, p1  EM, p3  EM, p6 | 1.42 x10^-03^ (3.62 x10^-03^, 6.86 x10^-04^)  8.12 x10^-04^ (1.76 x10^-03^, 4.62 x10^-04^)  2.55 x10^-04^ (9.32 x10^-04^, 1.01 x10^-04^) |
|  | EM | 1.87 x10^-04^ (1.16 x10^-03^, 2.37 x10^-06^) |
|  | DM | 1.19 x10^-03^ (4.32 x10^-03^, 7.03 x10^-05^) |
|  | Discs | 1.26 x10^-04^ (1.70 x10^-04^, 3.34 x10^-05^) |
| CYP3A22 | EM, p1  EM, p3  EM, p6 | 7.97 x10^-03^ (2.34 x10^-02^, 1.71 x10^-03^)  9.94 x10^-03^ (8.17 x10^-02^, 3.00 x10^-03^)  1.02 x10^-02^ (4.05 x10^-02^, 4.44 x10^-03^) |
|  | EM | 1.65 x10^-03^ (1.52 x10^-02^, 2.76 x10^-05^) |
|  | DM | 2.56 x10^-02^ (6.35 x10^-02^, 7.02 x10^-03^) |
|  | Discs | 8.85 x10^-03^ (1.57 x10^-02^, 8.06 x10^-04^) |
| HNF4a | EM, p1  EM, p3  EM, p6 | 3.07 x10^-02^ (9.79 x10^-02^, 1.48 x10^-02^)  4.26 x10^-02^ (1.10 x10^-01^, 2.22 x10^-02^)  2.63 x10^-02^ (6.18 x10^-02^, 8.73 x10^-03^) |
|  | EM | 1.94 x10^-02^ (4.06 x10^-02^, 1.10 x10^-02^) |
|  | DM | 5.10 x10^-02^ (1.22 x10^-01^, 3.82 x10^-02^) |
|  | Discs | 5.31 x10^-02^ (1.99 x10^-01^, 1.48 x10^-02^) |
| TF | EM, p1  EM, p3  EM, p6 | 1.29 x10^-02^ (2.76 x10^-01^, 5.12 x10^-03^)  4.88 x10^-02^ (2.46 x10^-01^, 3.06 x10^-02^)  2.27 x10^-02^ (6.22 x10^-02^, 9.58 x10^-03^) |
|  | EM | 1.34 x10^-02^ (6.59 x10^-02^, 6.50 x10^-05^) |
|  | DM | 5.85 x10^-03^ (1.53 x10^-02^, 1.68 x10^-03^) |
|  | Discs | 5.82 x10^-04^ (1.45 x10^-03^, 9.38 x10^-05^) |
| FAH | EM, p1  EM, p3  EM, p6 | 2.29 x10^-02^ (3.94 x10^-02^, 1.24 x10^-02^)  3.42 x10^-02^ (9.93 x10^-02^, 2.42 x10^-02^)  1.38 x10^-02^ (4.61 x10^-02^, 0.00 x10^+00^) |
|  | EM | 1.77 x10^-02^ (2.76 x10^-02^, 1,25 x10^-02^) |
|  | DM | 2.51 x10^-02^ (3.70 x10^-02^, 1.09 x10^-02^) |
|  | Discs | 1.65 x10^-02^ (6.95 x10^-01^, 9.44 x10^-03^) |
| TTR | EM, p1  EM, p3  EM, p6 | 6.12 x10^-05^ (1.07 x10^-04^, 1.85 x10^-05^)  3.75 x10^-05^ (1.33 x10^-04^, 2.09 x10^-05^)  3.80 x10^-05^ (2.98 x10^-04^, 2.82 x10^-05^) |
|  | EM | 7.56 x10^-06^ (6.52 x10^-05^, 8.73 x10^-07^) |
|  | DM | 2.00 x10^-04^ (9.31 x10^-04^, 5.32 x10^-05^) |
|  | Discs | n/a |

Supplementary Table 2: Medium composition Expansion and Differentiation Medium

| Component | Concentration in  Expansion Medium (EM) | Concentration in  Differentiation Medium (DM) |
| --- | --- | --- |
| Advanced DMEM/F12 | | |
| Glutamax | 1% (v/v) | 1% (v/v) |
| Penicillin-Streptomycin | 1% (v/v) | 1% (v/v) |
| HEPES | 1% (v/v) | 1% (v/v) |
| Wnt3a-conditioned medium | 30% (v/v) | - |
| N2 | 1% (v/v) | 1% (v/v) |
| Nicotinamide | 10 mM | - |
| B27 without vitamin A | 2% (v/v) | 2% (v/v) |
| R-spondin conditioned medium | 10% (v/v) | - |
| N-acetylcysteine | 1.25 mM | 1.25 mM |
| Y-27632 | 10 μM | - |
| A83-01 | 5 μM | 5 μM |
| Human epidermal growth factor (EGF) | 50 ng/mL | 50 ng/mL |
| Human noggin | 0.1 μg/mL | 0.1 μg/mL |
| Fibroblast growth factor 10 (FGF10) | 0.1 μg/mL | - |
| Gastrin | 10 nM | 10 nM |
| Hepatocyte growth factor (HGF) | 25 ng/mL | 25 ng/mL |
| Forskolin | 10 μM | - |
| Primocin | 1% (v/v) | 1% (v/v) |
| FGF19 | - | 0.1 μg/mL |
| Dexamethasone | - | 30 μM |
| DAPT (y-secretase inhibitor) | - | 10 μM |
| BMP7 | - | 25 ng/mL |


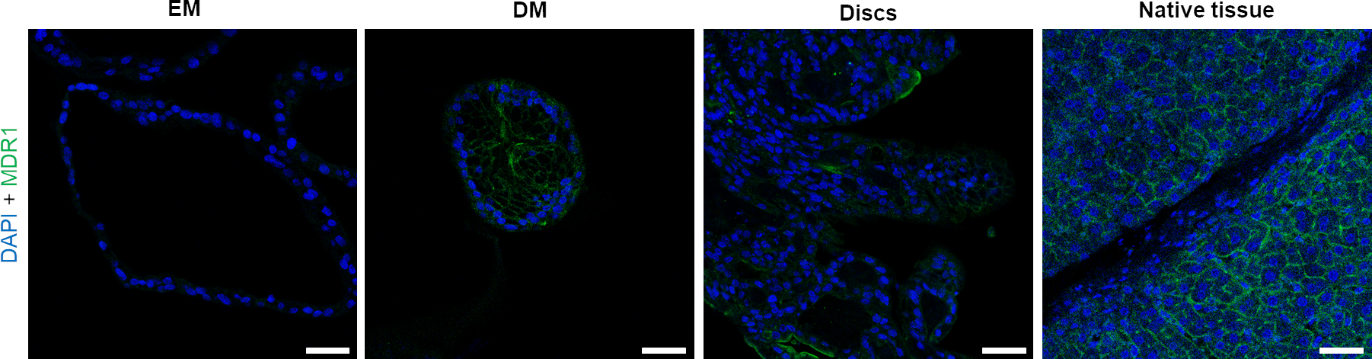


Supplementary Figure 1: Representative immunofluorescent staining for nuclei (DAPI in blue) and polarization marker MDR1 (Multidrug resistance protein 1) (all in green) for organoids in EM (Expansion Medium) and DM (Differentiation Medium) conditions, recellularized discs after day 5 and native liver tissue. MDR1 shows polarization of organoids in DM conditions and on discs. Scale bars represent 25 μm.
